# Novel mutations in Darier disease and association to self-reported disease severity

**DOI:** 10.1371/journal.pone.0186356

**Published:** 2017-10-13

**Authors:** Ivone U. S. Leong, Alexander Stuckey, Tara Ahanian, Martin Cederlöf, Jakob D. Wikstrom

**Affiliations:** 1 Dermatology and Venereology Unit, Department of Medicine (Solna), Karolinska Institutet, Stockholm, Sweden; 2 Division of Gene Technology, School of Biotechnology, Royal Institute of Technology, Science for Life Laboratory, Stockholm, Sweden; 3 Dermato-Venereology Clinic, Karolinska University Hospital, Stockholm, Sweden; 4 Department of Medical Epidemiology and Biostatistics, Karolinska Institutet, Stockholm, Sweden; Ohio State University Wexner Medical Center, UNITED STATES

## Abstract

Darier disease is a rare and severe autosomal dominant skin disease characterised by malodorous keratotic papules in seborrheic areas of the skin. Darier disease affects up to 1 in 30 000 people and is caused by mutations in the *ATP2A2* gene, which encodes to the sarco/endoplasmic reticulum calcium-ATPase isoform 2 that pumps calcium into the endoplasmic reticulum. Although many *ATP2A2* variants have been described, it is not known if genotype correlates with phenotype, which could be important for prognosis and treatment. This is the first study to use whole exome sequencing to screen the *ATP2A2* gene in a cohort of 28 clinically diagnosed Darier disease patients. Twenty-one different disease causing variants were identified and 15 of these were novel. Sixteen of the 21 variants were predicted to be pathogenic using *in silico* prediction programs. There were seven missense, four intronic/splice-sites, three frameshifts, two in-frame deletions, four nonsense and one synonymous mutations. This study also found ten patients who harbour more than one *ATP2A2* variant. The phenotype of the patient cohort was assessed by photography and by patient questionnaires. The genotype-phenotype association was examined for all variants in relation to the patient’s disease severity score, and no correlation could be established.

## Introduction

Darier disease (DD), also known as keratosis follicularis, is a rare and severe autosomal dominant skin disease, estimated to affect 1 in 30 000 to 100 000 people [1, 2]. The disease is characterized by keratotic papules and malodorous plaques in seborrheic areas of the skin, which can lead to large, crusted plaques. The disease often emerges during childhood and continues throughout adolescence, and can negatively influence the quality of life [2]. Disease phenotype is highly variable; even when two family members are affected by the same variant, the clinical symptoms may be different [3], which makes genotype-phenotype correlations difficult to determine. UVB irradiation, heat, friction and infections in affected areas can further exacerbate symptoms [4]. Other non-dermatological symptoms of DD include psychiatric conditions, such as intellectual disability and bipolar disease [2, 5].

**Fig 1:**
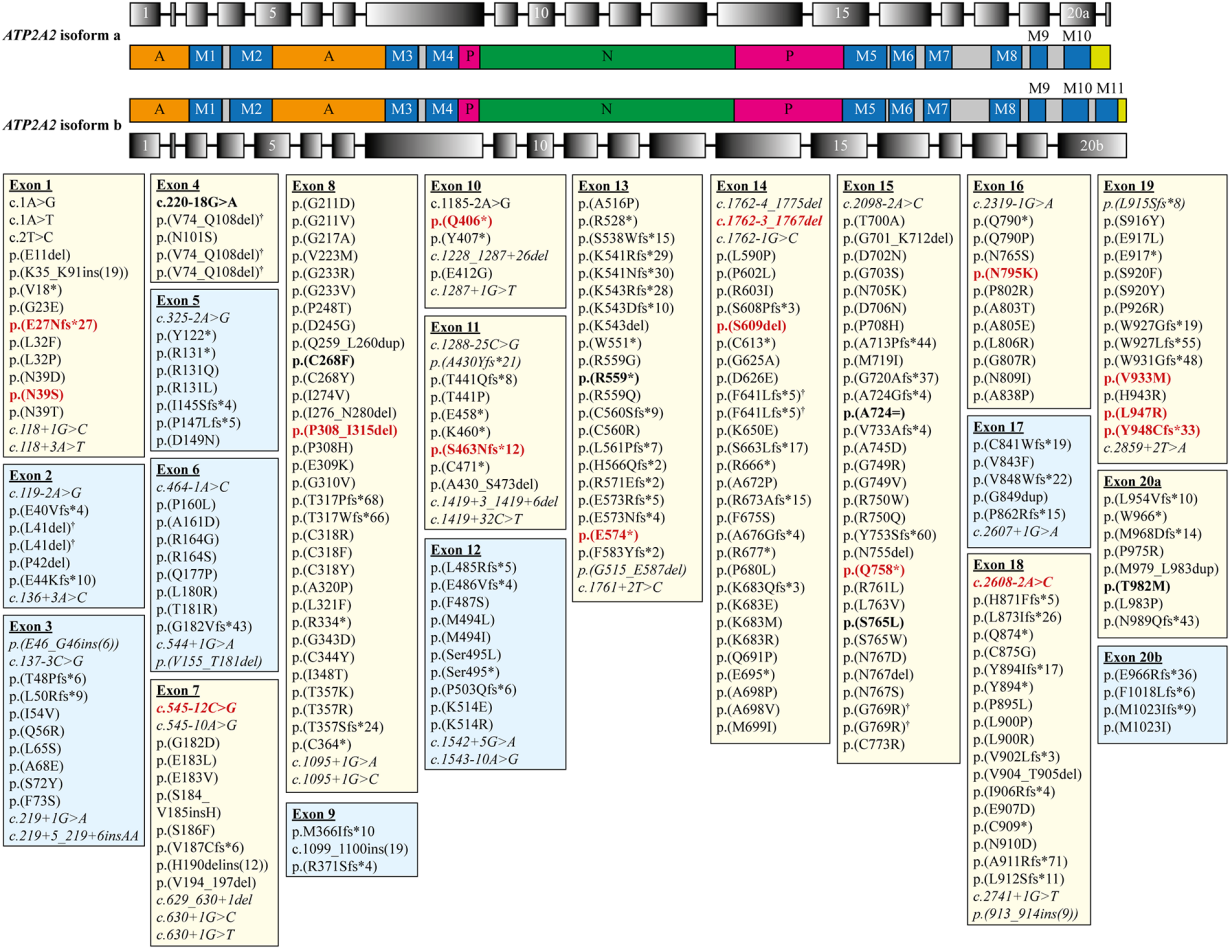
The location of all published/reported *ATP2A2* variants (in both isoforms) and variants discovered in the current study associated with Darier disease. *ATP2A2* isoform a consists of 21 exons (top row), which encodes for 10 transmembrane domains (M1 –M10, second row), and it is expressed in cardiomyocytes and slow-tiwtch skeletal muscles. The *ATP2A2* isoform b consists of 20 exons (fourth row), which encodes for 11 transmembrane domains (M1 –M11, third row) and it is expressed in most tissue types [3]. Both isoforms have an actuator domain (A, orange boxes), phosphorylation domain (P, pink boxes), nucleotide binding domain (N, green boxes) and C-terminus (lime green boxes). All genetic variants are listed in either pale yellow (exon with variants discovered in this study) or blue boxes (exon with no variants from this study). Novel genetic variants found in this study are in bold and coloured red. Variants that were discovered in this study but were not novel are in bold only. Intronic variants are in italics. † indicates *ATP2A2* variants with different genetic changes, but resulted in the same amino acid change.

DD is caused by mutations in the *ATP2A2* gene, which encodes the sarco/endoplasmic reticulum calcium (Ca^2+^) -ATPase isoform 2 (SERCA2), a calcium pump in the endoplasmic reticulum (ER) [6]. The *ATP2A2* gene transcript has two isoforms (isoform a and b), and consists of 21 or 20 exons, respectively (Fig 1). Both SERCA2 isoforms have a phosphorylation (P), nucleotide-binding domain (N) and the actuator domain (A; Fig 1).

Currently, there are over 270 unique *ATP2A2* mutations associated with DD [7, 8], and there are no mutational hotspots. The genetic mutations are predominantly missense/nonsense mutations, followed by deletions, splice-site and insertion mutations. Studies have shown that mutations in the *ATP2A2* gene disrupt normal SERCA2 protein function and leads to impaired/loss of Ca^2+^ transport in the ER, which leads to decreased ER Ca^2+^ stores [9–14]. Ca^2+^ imbalance affects cell-to-cell adhesion in skin and this could explain the DD skin phenotype [15].

To date, there has been no successful genotype-phenotype correlation established in DD. However, this could be important for the diagnosis of disease severity prediction and prognosis of patients. While an epidemiological study of intellectual disability and cognitive ability in Swedish DD patients have been conducted [16], the genetic aspect and the genotype-phenotype association have not been investigated in Sweden. In the current study, the coding sequence of *ATP2A2* of a cohort of 28 clinically diagnosed Swedish DD patients was screened using whole exome sequencing (WES). Patients were asked to score their disease severity and treatment efficacy. The genotype-phenotype association was also examined for all variants in relation to the patient’s disease severity score.

## Methods

### Patient recruitment

Twenty-eight patients with clinically diagnosed DD were recruited from the Dermato-Venereology clinic of Karolinska University Hospital and other Dermato-Venereology clinics in Sweden, including Uppsala, Linköping and Uddevalla. All patients were Caucasian.

### Ethical disclosure

All patients (and/or patients’ guardians) gave written informed consent for genetic testing.

This study was approved by the Stockholm regional ethical board (2017/1098-32).

### Patient questionnaire and determination of total body surface area

A questionnaire based on the study conducted by Burge and Wilkinson, (1992) [2] was issued to the patient cohort. The questions included the age of disease onset, the type of medication currently taken, the medication effect (ranked 1–5), disease severity (mild/moderate/severe and ranked from 1–5), general skin symptoms, quality of life, and factors that worsen disease (heat/friction/menstruation/pregnancy/stress/sun). The scores from 1–5 correspond to the following: 1, bad; 2, acceptable; 3, good; 4, very good; and 5, excellent [17].

The total body surface area (TBSA) affected by disease was assessed for each patient using photographs taken at the time when blood was drawn for WES. The TBSA was based on the rule of nines, which is a method used to quantify the area of affected skin in burns victims [18]. The assessment was blinded in that the genotype was unknown to the examiner.

### Whole exome sequencing and bioinformatics analysis

The Centre of Genomics and Transcriptomics (CeGAT, Tubingen, Germany) performed the WES. The exome was enriched by the Agilent SureSelectXT Human All Exon V6 (Agilent, CA, USA). The enriched libraries were sequenced using the Illumina HiSeq (Illumina, CA, USA) high-throughput sequencing platform with a read-length of 2 x 100 bp. Raw sequencing reads were processed according to the CeGAT bioinformatics streamlined process to generate a list of *ATP2A2* gene variants.

### Bidirectional Sanger-based sequencing confirmation

All reported *ATP2A2* variants were confirmed by bi-directional Sanger-based sequencing. All *ATP2A2* PCR primers were previously published by Ringpfeil et al., (2001) [19], with the addition of M13 forward or reverse sequences on the 5’ end. PCR was performed as previously described [20]. PCR products were electrophoresed in 1% UltraPure^™^ Agarose (Thermo Fisher Scientific, MA, USA) with GelRed^™^ (Biotium, CA, USA) incorporated into the gel.

Subsequent to gel electrophoresis confirmation, the PCR products were submitted to KI Gene (Centre for Molecular Medicine, Karolinska University Hospital Solna, Sweden) for bi-directional Sanger-based sequencing. The sequence analysis was performed using Geneious software (version 7.1.9) [21].

### *ATP2A2* variant analysis

The pathogenicity of missense and intronic/splice-site variants were assessed using *in silico* prediction programs. Missense variants were assessed with PolyPhen-2 [22], SIFT [23] and SNPs&GO [24], and the splice-site variants were assessed with ASSP [25] and HSF [26]. A mutation is categorised as benign or pathogenic when the majority of scores from the prediction programs are concordant [27]. For nonsense and frameshift mutations, it was assumed that the variants would be deleterious to correct protein function. The variants were not categorised solely based on the predicted outcomes; dbSNP database (build 149) [28], Exome Aggregation Consortium (ExAC) database [29], and Exome Sequencing Project (ESP) [30] database were also used to determine if variants were rare polymorphisms.

## Results

Twenty-eight patients (19 females and nine males) with clinically diagnosed DD were screened for *ATP2A2* variants. Nine were family members from four different families (Table 1). Fourteen of the 28 patients have a family history of DD, nine could be sporadic cases and two patients were adopted (Table 1). Twenty-one different variants were found, and 15 were novel variants that have not been previously reported in the literature or any databases (i.e. dbSNP [28] or LOVD database [7, 8]; Table 1 and Fig 1).

**Table 1:** List of *ATP2A2* mutations found in DD patients described in this study.

ID	Age of testing	Age of onset	Sex	Family history of disease	Nucleotide change	Amino acid change	Zygosity	Exon	Domain	Mutation type	dbSNP	ExAC/ESP	Predicted outcome	Reported
1	43		F											
2	27	11	M	Yes	c.2840T>G	p.L947R	Het	19	M 9	Missense			Pathogenic	
3	50	19	F	No										
4	51	14	M	No	c.1762-3_1767del		Het	14	N	Essential splice-site			Pathogenic	
5*	47	19	M	No	c.2172G>A	p.A724=	Het	15	P	Synonymous	rs56243033	0.055/0.05	Benign	[31]
6	56	3.5	M	Yes	c.2840T>G	p.L947R	Het	19	M 9	Missense			Pathogenic	
7^†^	68	20	F	Yes	c.1675C>T	p.R559*	Het	13	N	Nonsense			Pathogenic	
8	67	64	F	Adopted	c.220-18G>A		Het	i3	M 1	Intronic	rs35235621	0.015/0.018	Benign	
					c.2843_2844delAT	p.Y948Cfs*33	Het	19	M 9	Frameshift			Pathogenic	
9^†^	30		M		c.1675C>T	p.R559*	Het	13	N	Nonsense			Pathogenic	
					c.2172G>A	p.A724=	Het	15	P	Synonymous	rs56243033	0.055/0.05	Benign	[31]
10	44	14	F	Adopted	c.1216C>T	p.Q406*	Het	10	N	Nonsense			Pathogenic	
					c.2172G>A	p.A724=	Het	15	P	Synonymous	rs56243033	0.055/0.05	Benign	[31]
11	60	12	F	Yes	c.2608-2A>C		Het	i17		Essential splice-site			Pathogenic	
12	64		F	Yes	c.2608-2A>C		Het	i17		Essential splice-site			Pathogenic	
13	78	15	F	Yes	c.803G>T	p.C268F	Het	8	M 3	Missense	rs121912733		Pathogenic	[3]
					c.1386_1390delTTCTA	p.S463Nfs*12	Het	11	N	Frameshift			Pathogenic	
14	67	16	M	Yes	c.922_945del	p.P308_I315del	Het	8	M 4	Deletion (in-frame)			Likely pathogenic	
15**	65	22.5	F	Yes	c.79delG	p.E27Nfs*27	Het	1	A	Frameshift			Pathogenic	
16**	44	14	F	Yes	c.79delG	p.E27Nfs*27	Het	1	A	Frameshift			Pathogenic	
17	60	20	F	No	c.2945C>T	p.T982M	Het	20	M 10	Missense	rs149024535	0.0017/0.0003	Benign	[32]
18	81	Unsure	F	No	c.2294C>T	p.S765L	Het	15	M 5	Missense			Pathogenic	
19	61	16	F	No	c.220-18G>A		Het	i3	M 1	Intronic	rs35235621	0.015/0.018	Benign	
					c.1825_1827delTCC	p.S609del	Het	14	P	Deletion (in-frame)	rs767885880	0.000008	Likely pathogenic	
20	49	13	F	No	c.545-12C>G		Het	i6	A	Intronic			Benign	
					c.2797G>A	p.V933M	Het	19	M 9	Missense	rs372102705	0.0001/0.00008	Benign	
21	55	14	F	Yes	c.2385T>G	p.N795K	Het	16	M 6	Missense			Pathogenic	
22*	48	20	F	Yes	c.1720G>T	p.E574*	Het	13	N	Nonsense			Pathogenic	
					c.2172G>A	p.A724=	Het	15	P	Synonymous	rs56243033	0.055/0.05	Benign	[31]
23*	79	23	F	Yes	c.1720G>T	p.E574*	Het	13	N	Nonsense			Pathogenic	
					c.2172G>A	p.A724=	Hom	15	P	Synonymous	rs56243033	0.055/0.05	Benign	[31]
24	33	22	F	Yes	c.116A>G	p.N39S	Het	1	A	Missense			Pathogenic	
					c.220-18G>A		Het	i3	M 1	Intronic	rs35235621	0.015/0.018	Benign	
25	56	15	M	No										
26^††^	66	16	F	Yes	c.2272C>T	p.Q758*	Het	15	M 5	Nonsense			Pathogenic	
27^††^	32		M		c.2272C>T	p.Q758*	Het	15	M 5	Nonsense			Pathogenic	
					c.2172G>A	p.A724=	Het	15	P	Synonymous	rs56243033	0.055	Benign	[31]
28	61	20	M	No										

*^, †,^ **^, ††^ represent patients that are related. Rows that are blank are patients who did not harbour an *ATP2A2* variant. F, female; M, male; Het, heterozygous; Hom, homozygous; i*x*, intron *x*; M*x*, transmembrane domain *x*; A, actuator domain; P, phosphorylation domain; N, nucleotide binding domain.

Four patients (patients 1, 3, 25 and 28) did not have any variants in the *ATP2A2* gene despite clinically diagnosed with DD, and two patients had benign *ATP2A2* variants (patient 5 and 17, Table 1). Ten patients had two different *ATP2A2* variants, and for most of these patients, one variant was benign and the other predicted to be pathogenic (Table 1). One patient had two benign variants (Patient 20), and one patient had two pathogenic variants (Patient 13; Table 1). The read-depth of each of the *ATP2A2* exons for each patient sample is shown in S1 Fig.

### Spectrum of variants

Sixteen of 21 variants were predicted to be pathogenic (Table 1 and S1 and S2 Tables). There are five missense mutations; two in-frame deletions; two essential splice-site mutations; three frameshift mutations and four nonsense mutations (Table 1). The benign variants consisted of two intronic mutations, two missense mutations, and one synonymous mutation (Table 1). All variants are scattered throughout *ATP2A2* and these are shown in Fig 1 in relation to all previously reported *ATP2A2* variants [7, 8]. Eleven variants are located in the transmembrane domains (M1, M3, M4, M5–7, M9 and M10), three in the actuator (A) domain, five in the nucleotide binding (N) domain, and two in the phosphorylation (P) domain (Table 1).

### Phenotypic analysis

To investigate whether there is a genotype-phenotype correlation, patients were asked a series of questions regarding their disease state. Twenty-five of the 28 patients (89%) responded, 18 women and seven men. Four of these patients did not harbour an *ATP2A2* genetic variant.

**Fig 2:**
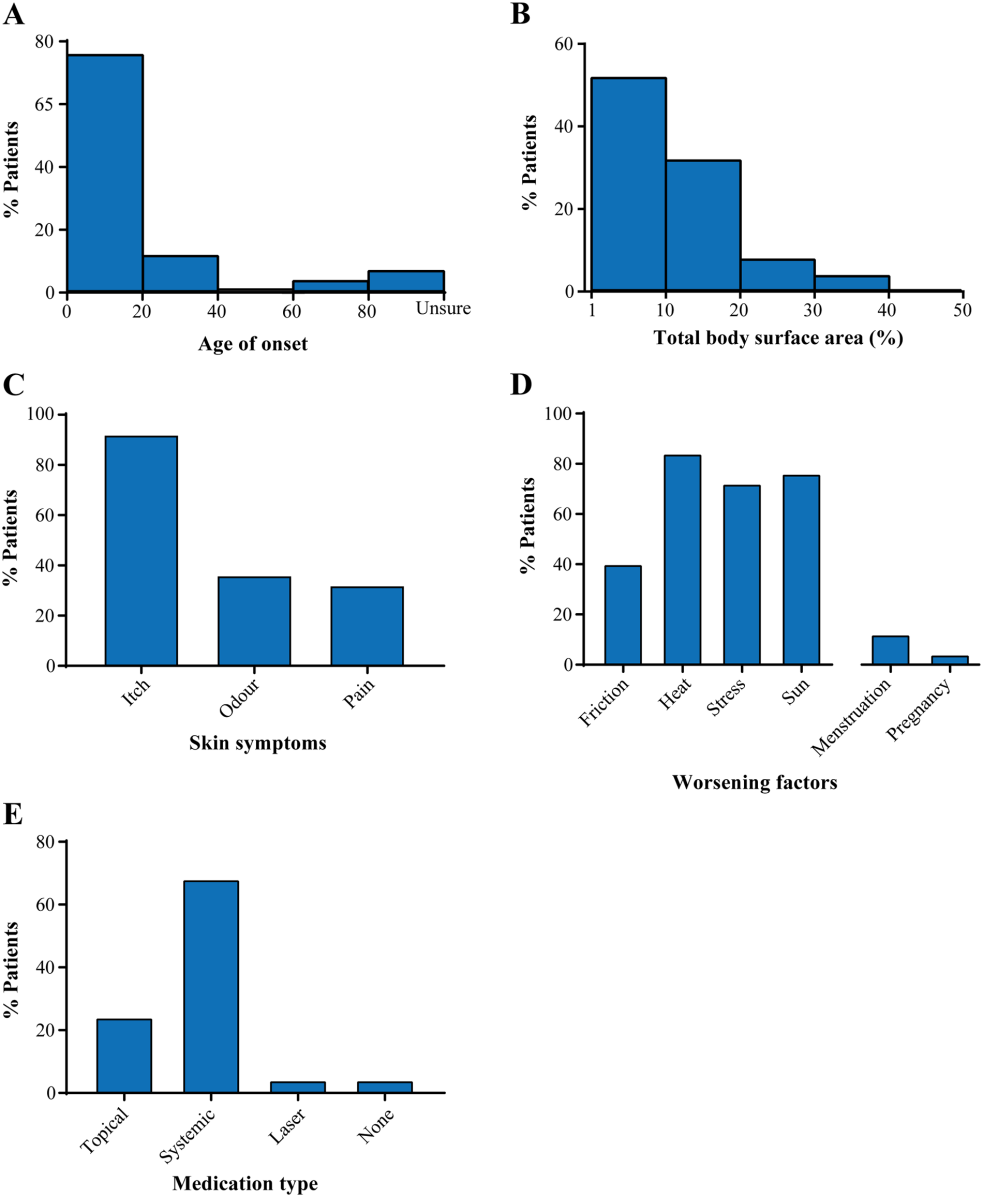
Clinical characteristics of the cohort. Age of onset (A), total body surface area affected (B), skin symptoms (C), worsening factors (D) and medication type (E) in the patient cohort.

The age of onset for the majority of patients was below 20 years of age (76%; Fig 2A). The total body surface area (TBSA) affected by disease was also variable, with the majority of patients having disease on 1–20% of their bodies (84%; Fig 2B). The most commonly affected skin areas were the skin folds (56%) and trunk of the body (52%; Table 2). Itchy skin (92%), heat, sun and stress (72–84%) were the most common exacerbating factor for many patients (Fig 2C and 2D). More than half of the patients who responded to the questionnaire (60%) were negatively affected by DD and found that it affected their life quality (Table 2). Most patients were treated systemically with either acitretin or isotretionin (68%), and some were treated topically (24%; Fig 2E).

**Table 2:** Patient clinical characteristics.

ID	Sex	Nucleotide change	Type of medication	TBSA (%)	Affected skin areas	Reduction in quality of life?	General skin symptoms	Factors that worsens disease
1	F							
2	M	c.2840T>G	Moisturizer	18	Face, scalp, trunk, only mildly in skin folds, mild elsewhere	Yes	Itch	Friction, heat, friction, stress, sun
3	F		Topical		Back, shoulders, trunk	No	Itch	Heat, sun
4	M	c.1762-3_1767del	Acitretin	4	Face, scalp, trunk, only mildly in skin folds	No	Itch	Heat, stress, sun
5*	M	c.2172G>A	Topical	1	Trunk, shoulders, back	No	Itch	Heat, stress, sun
6	M	c.2840T>G	Acitretin	10	Back, shoulders, trunk	No	Itch	Stress
7^†^	F	c.1675C>T	Acitretin	1	Small defined area only on one side	No	Itch	Heat, sun
8	F	c.220-18G>A	Acitretin	5	Trunk	No	NA	Sun
		c.2843_2844delAT						
9^†^	M	c.1675C>T						
		c.2172G>A						
10	F	c.1216C>T	Isotretinoin	6	Trunk	Yes	Itch	Heat, sun
		c.2172G>A						
11	F	c.2608-2A>C	Acitretin	17	Hands, feet	Yes	Itch, pain	Friction, heat, stress, sun
12	F	c.2608-2A>C	Acitretin	12	Face, folds, trunk	Yes	Itch	Friction, heat, stress, sun
13	F	c.803G>T	Acitretin	18	Face, hands, scalp	Yes	Odour, pain	Friction, heat, menstruation, pregnancy, stress, sun
		c.1386_1390delTTCTA						
14	M	c.922_945del	Acitretin	14	Folds, only mild elsewhere	No	Itch	Heat, stress, sun
15**	F	c.79delG	None	2	Folds, only mild elsewhere	Yes	Itch, odour, pain	Friction, heat, stress, sun
16**	F	c.79delG	Acitretin	12	Entire body	Yes	Itch, odour, pain	Friction, heat, stress
17	F	c.2945C>T	Laser	1	Face, scalp, trunk, only mildly in skin folds	Yes	Itch, odour	Heat
18	F	c.2294C>T	Acitretin	12	Lower back, knee folds, waist	No	Itch	Heat
19	F	c.220-18G>A	Acitretin	6	Back, hands, feet, thighs, trunk	Yes	Itch, odour, pain	Heat, stress, sun
		c.1825_1827delTCC						
20	F	c.545-12C>G	Acitretin	10	Face, scalp, trunk, only mildly in skin folds	Yes	Itch, pain	Friction, heat, menstruation, stress
		c.2797G>A						
21	F	c.2385T>G	Acitretin	22	Entire body, also pharynx	Yes	Itch, odour, pain	Friction, heat, stress, sun
22*	F	c.1720G>T	Isotretinoin	20	Folds, mild elsewhere	No	Itch	Heat, stress, sun
		c.2172G>A						
23*	F	c.1720G>T	Isotretinoin	25	Trunk	No	Itch	Heat, stress, sun
		c.2172G>A						
24	F	c.116A>G	Topical	1	Face, genital area, hands	Yes	Itch, odour, pain	Menstruation, stress
		c.220-18G>A						
25	M		Acitretin	38	Face, scalp, trunk, only mildly in skin folds	Yes	Itch	Friction, stress, sun
26^††^	F	c.2272C>T	Topical	2	Trunk, only mildly in skin folds, mild elsewhere	Yes	Itch, odour	Heat, sun
27^††^	M	c.2272C>T						
		c.2172G>A						
28	M		Topical	10	Scalp, trunk, only mildly in skin folds	Yes	Itch, pain	Friction, heat, friction, stress

*^, †,^ **^, ††^ represent patients that are related. Rows that are blank are patients who did not harbour an *ATP2A2* variant or they did not answer the questionnaire. TBSA, total body surface area; F, female; M, male; Het, heterozygous; Hom, homozygous; i*x*, intron *x*; M*x*, transmembrane domain *x*; A, actuator domain; P, phosphorylation domain; N, nucleotide binding domain.

**Fig 3:**
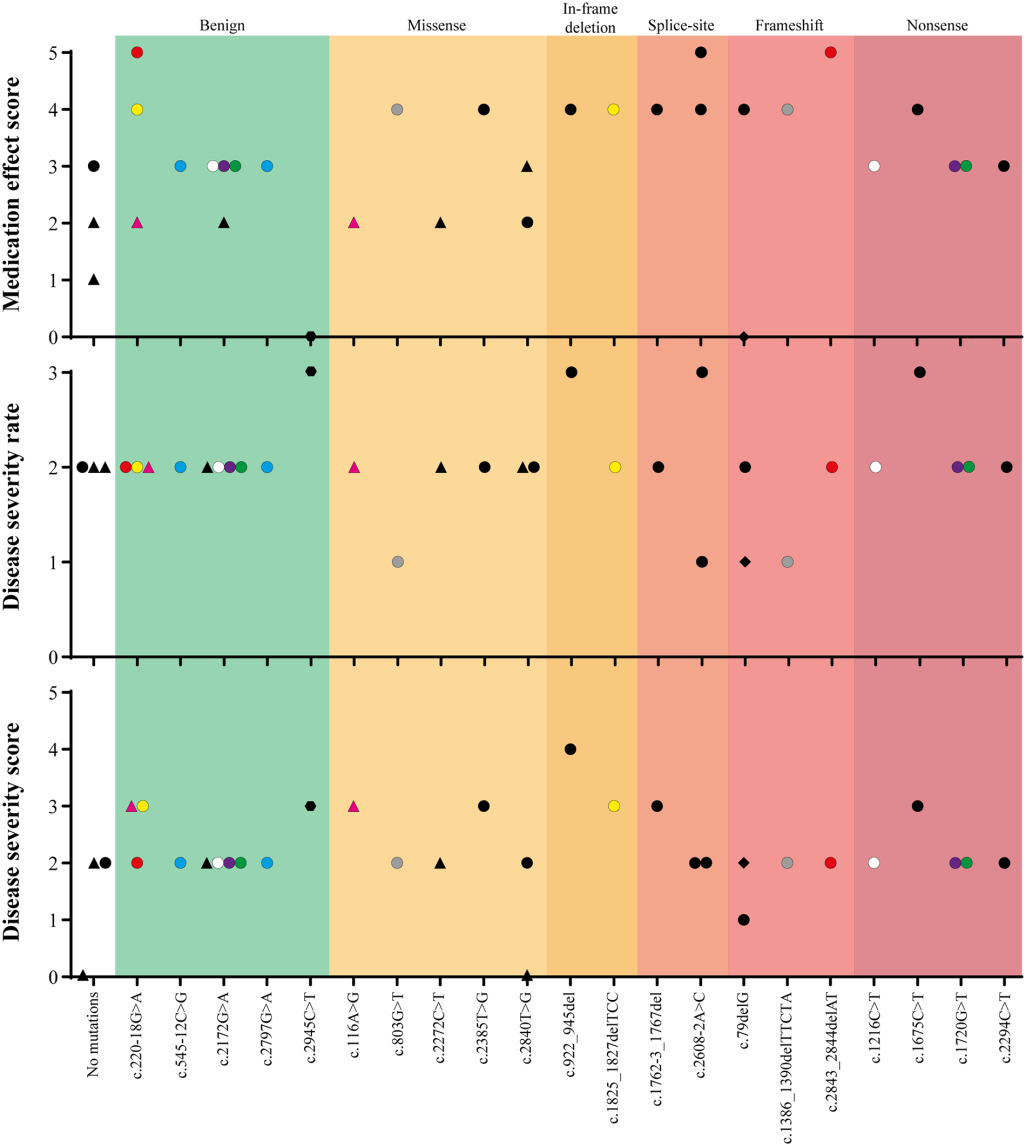
Medication effect (1–5), disease severity (mild–severe) and disease severity (1–5) scores sorted by mutation type. Medication effect and disease severity score (Top and bottom), a score of 1 = bad, 2 = acceptable, 3 = good, 4 = very good or 5 = excellent. Disease severity rate (Middle), a score of 1 = severe, 2 = moderate and 3 = mild. A score of 0 corresponds to patient’s lack of answer to a particular category. The type of *ATP2A2* mutation is shown on the x-axis, and they are sorted into seven categories: No mutations, benign, missense, in-frame deletion, splice-site, frameshift and nonsense mutations. ● represent patients receiving systemic treatment; ▲represents patients receiving topical treatment; ◆ represent patients receiving no treatment and 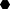 represent patients who have received laser treatment. The different coloured points represent the patients with two *ATP2A2* variants. Points with the same colours are variants in the same patient.

Patients were asked to score the medication effect and disease severity (Fig 3 and S2 Fig). Disease severity was scored in two different ways: whether disease was mild, moderate or severe; and ranked from 1–5 (1 corresponding to bad and 5 to excellent). Both medication effect and disease severity were analysed based on the *ATP2A2* variant and the protein domain location (S2 Fig), and the type of mutation (i.e. missense, frameshift, etc.; Fig 3).

Patients treated systemically were more satisfied with medication effect (good to excellent scores) than topically treated patients, who scored their medication effect as bad to good (Fig 3 and S2 Fig–top graphs). Genotype-negative patients scored their medication effects as bad to good, while benign variants scored medication effect as generally good (Fig 3, top graph). Medication effect for patients harbouring missense mutations were acceptable to very good regardless of treatment type (Fig 3, top graph). All patients with in-frame deletion, splice site, frameshift and nonsense mutations were systemically treated and ranked the medication effect at good to excellent (Fig 3, top graph).

The disease severity of genotype-negative patients or those with benign *ATP2A2* variants were rated as moderate (Fig 3, middle graph). Many patients scored disease severity at 2 (using the 1–5 scoring system), which equated to acceptable (Fig 3, bottom graph). Missense and in-frame deletion mutations caused mild to moderate disease, which is similar to the scores of 2 to 3 (acceptable to good) in disease severity (Fig 3, middle and bottom graphs). Splice-site and frameshift mutations scores were contradictory between the two different ranking systems. The scores varied widely for patients with either type of mutations (mild to severe); however, the corresponding disease severity scores ranged from 1 to 3 (bad to good).

## Discussion

To date, no study has successfully correlated genotype to phenotype in DD. In the current study, we screened a Swedish cohort of 28 clinically diagnosed DD patients using WES and found 15 novel variants that have not been reported previously. We estimate that this cohort comprises more than 10% of DD patients in Sweden. Genotype-phenotype correlation was investigated based on disease severity scores and failed to show mutations with specific clinical picture.

### Genotypic results

Twenty-one *ATP2A2* variants were identified in this study, 15 are novel variants that have not been previously reported. Sixteen of these variants are predicted to be pathogenic by *in silico* prediction programs. Despite the lack of functional studies investigating these variants, previous studies of different *ATP2A2* mutants (missense, nonsense and deletion mutations) showed that they all affect SERCA2 function by either decreasing protein expression, Ca^2+^-ATPase activity, Ca^2+^ transport or alter protein kinetic properties [9–14]. There is also evidence that mutant SERCA2 protein is an initiator of ER stress that causes human epidermal keratinocytes to round up, detach and induce apoptosis [10]. From these previous studies, it could be concluded that the 16 pathogenic variants reported in this study are likely to affect SRECA2 protein function. The variants are also associated with clinical data.

There were ten patients who had two *ATP2A2* variants (one double pathogenic and one double benign variants). To the authors’ best knowledge there has been no reported cases of patients harbouring two *ATP2A2* variants. Therefore, it is unknown whether the patient with two pathogenic variants have an additive effect on disease severity.

The four patients (14%) who are genotype-negative, but show skin symptoms could be wrongly diagnosed for DD. The number of genotype-negative patients in this study is similar to other reports in literature, which have a range of 7–33% of patients who are genotype-negative [8, 33–35]. Three of the four patients have rated their disease as moderately severe. The fact that no *ATP2A2* variants were detected by WES does not necessarily mean that there is no mutation in the gene. There could be large deletions/insertions or cryptic splice-sites present that could not be detected using WES and require other methods (i.e. comparative genomic hybridisation microarray–CGH arrays, or multiplex ligation-dependent probe amplification–MLPA) to detect the copy number changes. There is also the possibility that some variants are in the unscreened regions of the *ATP2A2* gene, such as the promoter regions, intronic regions or the 3’ untranslated region, which could affect expression/or function of the SERCA2 protein.

### Phenotypic/clinical results

The clinical features/symptoms of the current cohort are similar to previously described DD patients [2, 36], including age of onset, affected skin areas, skin symptoms and factors that exacerbate disease. The patients in this study are treated with either oral retinoids (acitretin or isotretinoin) or topical treatments, which are also typical for other DD patients [36]. Many patients have ranked the systemic treatment as effective against DD symptoms; however, the treatment is often not well tolerated and cannot be taken long term [2]. Like other reported DD cases, disease severity is varied [3, 19, 32, 35, 37–39]. Four patients in the current cohort ranked their disease as severe (16%), 18 as moderate (72%) and three as mild (12%, based on the disease severity rating results).

### Genotype-phenotype correlation

Similar to previous reports, establishing a cross-sectional genotype-phenotype correlation for DD was difficult [3, 19, 32, 35, 37–39]. Previous reports have shown that family members harbouring the same variant manifest different phenotypes [3], which was seen in the current study. Even when the *ATP2A2* variants were separated into protein region and mutation type (i.e. benign, missense, etc.), it was still difficult to find a correlation between genotype and phenotype. One possible reason could be that for a majority of *ATP2A2* variants, SERCA2 protein function is affected independent of the type or location of the mutation [9–14]. It could also be due to the variability of disease symptoms over time, which is influenced by environmental factors.

It is interesting to note that patients with only benign variants have moderate DD symptoms. Whether these variants have an effect on SERCA2 protein function is unknown, as none have been characterised. It is also possible that these patients could be DD phenocopies (non-genetic forms of the disease); however, Berg and Basset (1993) suggest the likelihood of this is rare [40]. This is because DD is a rare disease that is clearly inherited in an autosomal dominant manner [40]. There is also the possibility that the patients could harbour a second undetected *ATP2A2* variant (i.e. large deletion/insertion).

### Limitations

Currently, DD is diagnosed by its appearance and histopathology. There are some dermatological diseases that may also resemble DD. We believe that this “crude” non-molecular diagnostic method will be supported with genetic testing in the future to further confirm the clinical diagnosis. As well as analytical limitations that may not be detecting all DD variants, patients may be wrongly diagnosed with DD as their symptoms resemble DD, which confounds result interpretation. Other limitations of the study is that patients determined the medication efficacy and disease severity, and there will be variability in how these two factors were determined between patients. As the study only examined cross-sectional disease severity a longer and larger multicentre prospective study to investigate disease severity may be a better method of determining this factor.

## Conclusion

Despite being unable to establish a genotype-phenotype correlation as the disease severity is variable amongst patients, we identified 15 novel variants that have not been previously reported in DD. This study is the first to use WES to screen DD patients, and it is the first to investigate the genetic aspect in Swedish DD patients.

## Supporting information

S1 Fig The read-depth of all *ATP2A2* exons from all patient samples. An x represents each patient sample and yellow circles represent patients who are genotype-negative. The red bars show the median and 95% confidence intervals.
(TIF)

S2 FigThe scores patients gave for medication effect (1–5), disease severity (mild–severe) and disease severity (1–5) sorted by protein regions.Medication effect and disease severity score (Top and bottom), a score of 1 = bad, 2 = acceptable, 3 = good, 4 = very good or 5 = excellent. Disease severity rate (Middle), a score of 1 = severe, 2 = moderate and 3 = mild. A score of 0 corresponds to patient’s lack of answer to a particular category. The type of *ATP2A2* mutation is shown on the x-axis, and they are sorted into protein domains. ● represent patients receiving systemic treatment; ▲ represents patients receiving topical treatment; ◆ represent patients receiving no treatment and 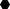 represent patients who have received laser treatment.(TIF)

S1 Table Polyphen-2, SNPs&GO and SIFT predictions for novel missense variants found in this study. (DOCX)

S2 Table ASSP and Human Splicing Finder predictions for novel variants affecting splice sites found in this study. (DOCX)
