# Supplementary figures and images for: Novel mutations in Darier disease and association to self-reported disease severity
Source: PLoS One. 2017 Oct 13;12(10):e0186356. doi: 10.1371/journal.pone.0186356 (PMC5640244; doi:10.1371/journal.pone.0186356)

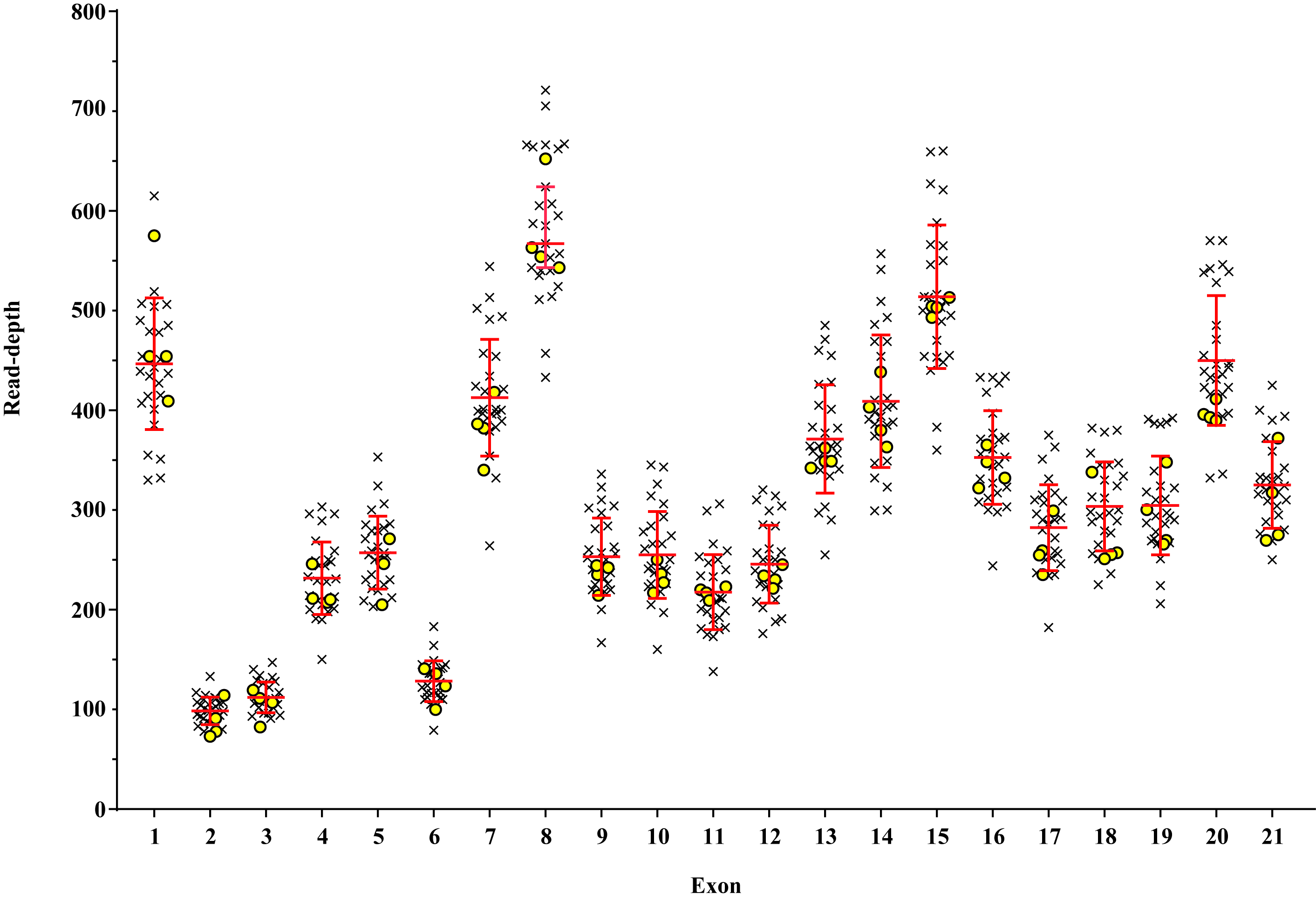

Supplement: S1 Fig — An x represents each patient sample and yellow circles represent patients who are genotype-negative. The red bars show the median and 95% confidence intervals. (TIF) [file pone.0186356.s001.tif]

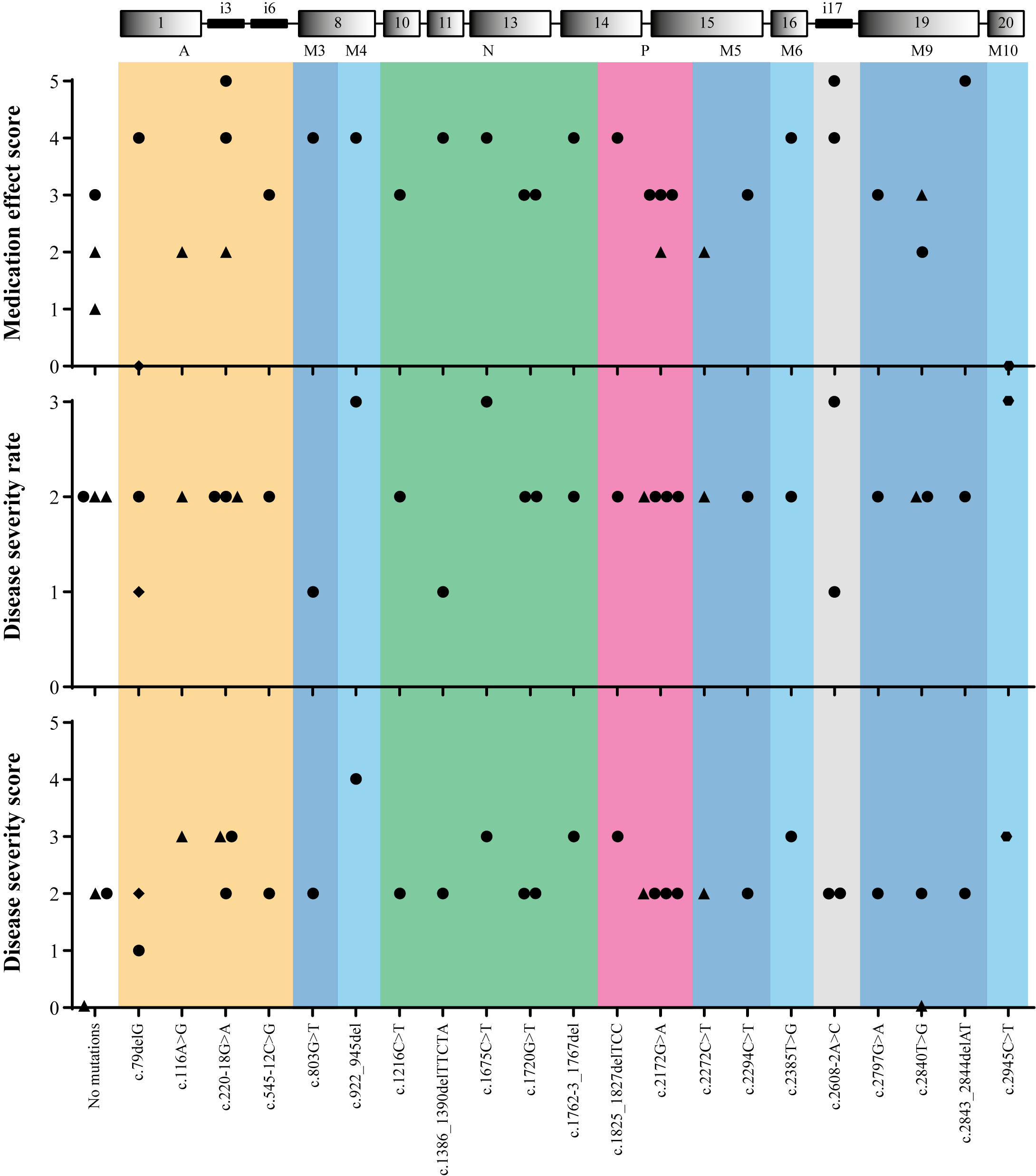

Supplement: S2 Fig — Medication effect and disease severity score (Top and bottom), a score of 1 = bad, 2 = acceptable, 3 = good, 4 = very good or 5 = excellent. Disease severity rate (Middle), a score of 1 = severe, 2 = moderate and 3 = mild. A score of 0 corresponds to patient’s lack of answer to a particular category. The type of ATP2A2 mutation is shown on the x-axis, and they are sorted into protein domains. ● represent patients receiving systemic treatment; ▲ represents patients receiving topical treatment; ◆ represent patients receiving no treatment and represent patients who have received laser treatment. (TIF) [file pone.0186356.s002.tif]
