# Supplementary material for: Novel mutations in Darier disease and association to self-reported disease severity
Source: PLoS One. 2017 Oct 13;12(10):e0186356. doi: 10.1371/journal.pone.0186356 (PMC5640244; doi:10.1371/journal.pone.0186356)
Supplement: S1 Table — (DOCX) [file pone.0186356.s003.docx]

**S1 table: Polyphen-2, SNPs&GO and SIFT predictions for novel missense variants found in this study**

| **Nucleotide change** | **Amino acid change** | **PolyPhen-2 results** | **SNPs&GO results** | **SIFT results** | **Overall results** |
| --- | --- | --- | --- | --- | --- |
| c.116A>G | p.N39S | Probably damaging | Disease | Damaging | Pathogenic |
| c.380G>A | p.G127D | Probably damaging | Disease | Damaging | Pathogenic |
| c.392G>A | p.R131Q | Probably damaging | Disease | Damaging | Pathogenic |
| c.803G>T | p.C268F | Probably damaging | Disease | Damaging | Pathogenic |
| c.2172G>A | p.A724= |  | Neutral | Tolerant | Benign |
| c.2294C>T | p.S765L | Probably damaging | Disease | Damaging | Pathogenic |
| c.2385T>G | p.N795K | Probably damaging | Disease | Damaging | Pathogenic |
| c.2797G>A | p.V933M | Benign | Neutral | Tolerant | Benign |
| c.2840T>G | p.L947R | Probably damaging | Neutral | Damaging | Pathogenic |
| c.2945C>T | p.T982M | Benign | Neutral | Tolerant | Benign |
