# Supplementary material for: Novel mutations in Darier disease and association to self-reported disease severity
Source: PLoS One. 2017 Oct 13;12(10):e0186356. doi: 10.1371/journal.pone.0186356 (PMC5640244; doi:10.1371/journal.pone.0186356)
Supplement: S2 Table — (DOCX) [file pone.0186356.s004.docx]

**S2 table: ASSP and Human Splicing Finder predictions for novel variants affecting splice sites found in this study**

| **Nucleotide change** | **ASSP results** | **Human Splicing Finder results** | **Overall results** |
| --- | --- | --- | --- |
| c.116A>G | Abolished splice site | Introduced new splice acceptor site | Affects splicing |
| c.220-18G>A | No change | No change | Benign |
| c.545-12C>G | No change | No change | Benign |
| c.1762-3_1767del | Abolished splice site | Abolished splice site | Affects splicing |
| c.2608-2A>C | Abolished splice site | Abolished splice site | Affects splicing |
